# On the Thermal and Thermodynamic (In)Stability of Methylammonium Lead Halide Perovskites

**DOI:** 10.1038/srep31896

**Published:** 2016-08-22

**Authors:** Bruno Brunetti, Carmen Cavallo, Andrea Ciccioli, Guido Gigli, Alessandro Latini

**Affiliations:** 1 Consiglio Nazionale delle Ricerche - Istituto per lo Studio dei Materiali Nanostrutturati, c/o Dipartimento di Chimica, Università degli Studi di Roma “La Sapienza”, Piazzale Aldo Moro 5, 00185 Roma, Italy; 2 Dipartimento di Chimica, Università degli Studi di Roma “La Sapienza”, Piazzale Aldo Moro 5, 00185 Roma, Italy

## Abstract

The interest of the scientific community on methylammonium lead halide perovskites (MAPbX_3_, X = Cl, Br, I) for hybrid organic-inorganic solar cells has grown exponentially since the first report in 2009. This fact is clearly justified by the very high efficiencies attainable (reaching 20% in lab scale devices) at a fraction of the cost of conventional photovoltaics. However, many problems must be solved before a market introduction of these devices can be envisaged. Perhaps the most important to be addressed is the lack of information regarding the thermal and thermodynamic stability of the materials towards decomposition, which are intrinsic properties of them and which can seriously limit or even exclude their use in real devices. In this work we present and discuss the results we obtained using non-ambient X-ray diffraction, Knudsen effusion-mass spectrometry (KEMS) and Knudsen effusion mass loss (KEML) techniques on MAPbCl_3_, MAPbBr_3_ and MAPbI_3_. The measurements demonstrate that all the materials decompose to the corresponding solid lead (II) halide and gaseous methylamine and hydrogen halide, and the decomposition is well detectable even at moderate temperatures (~60 °C). Our results suggest that these materials may be problematic for long term operation of solar devices.

In 2009, the pioneering work published by Kojima *et al.*^1^ regarding the use of methylammonium lead halide perovskites as sensitizers in solar cells, though obtaining quite modest results in comparison to then established dye-sensitized solar cells-DSSCs (solar conversion efficiencies obtained: 3.13% and 3.89%, with MAPbBr_3_ and MAPbI_3_, respectively vs. 11.2% for the best performing DSSC that year^2^), sparked an enormous and well motivated interest of the scientific community about this new class of sensitizers. In fact, the optimization of the device led to a very quick performance improvement of the same and hybrid cells based on MAPbX_3_ sensitizers soon outperformed their DSSC counterpart. In fact, as of 2015, the best performing perovskite cells attained solar conversion efficiency of 20.1%^3^ vs 13% of the best DSSC^4^. But this stunning development is accompanied by many problems^5^,^6^,^7^ the most important being related to hysteresis of cells and stability issues of MAPbX_3_ compounds with regards to interaction with atmospheric agents (especially moisture)^7^ and to their thermal and thermodynamic stability towards decomposition, especially in severe operating conditions such as under intense solar irradiation. Despite the extreme importance of information regarding the last two issues, i.e. thermal and thermodynamic stability towards decomposition of the materials for the technological development of solar devices based on MAPbX_3_ compounds, the available literature does give only very limited experimental information^8^,^9^,^10^,^11^.

Here we present the results of two parallel investigations on MAPbCl_3_, MAPbBr_3_ and MAPbI_3_.

The first is a study of the thermal stability of the compounds by means of non-ambient X-ray diffraction.

The second is a study of the thermodynamics of decomposition of the compounds by Knudsen effusion-mass spectrometry (KEMS) and Knudsen effusion mass loss (KEML) techniques.

By the combination of these two studies, we clarify for the first time the decomposition reactions and we obtain both kinetic data useful for the estimation of the lifetime of the devices in operative conditions as well as thermodynamic data necessary to assess the stability of the compounds in diverse conditions, such those encountered during the realization of the devices and during their operation.

## Thermal Stability: Non-Ambient X-ray Diffraction

**Figure 1:**
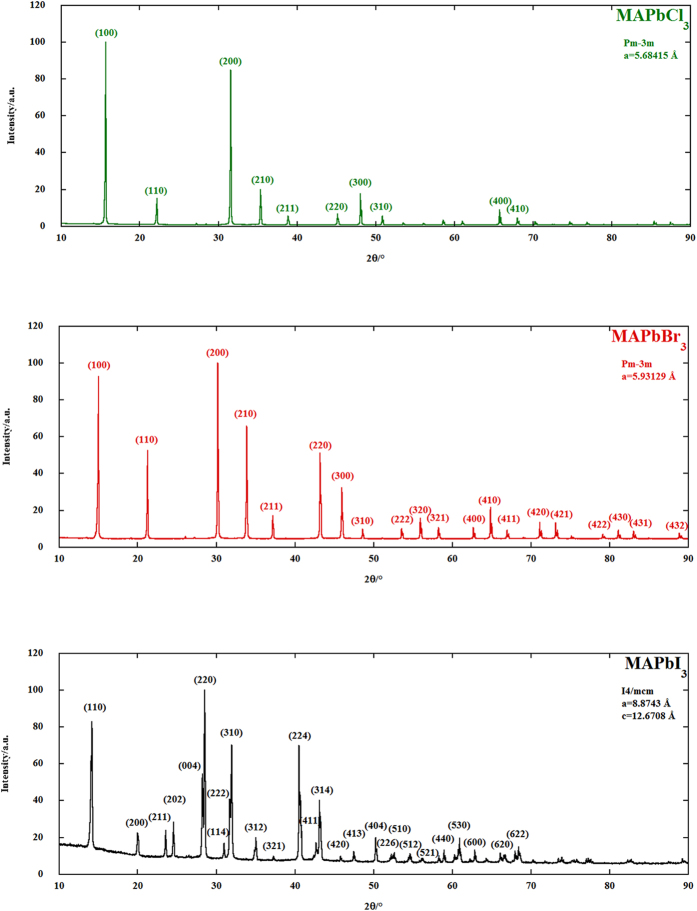
X-ray diffraction patterns of the synthesized MAPbX_3_ compounds. All the diffraction patterns show only the reflections of the perovskite phases, with no lead (II) halides detectable.

Conventional powder X-ray diffraction has been used to check the phase purity of the synthesized compounds. The diffractograms of MAPbCl_3_, MAPbBr_3_ and MAPbI_3_ are presented in Fig. 1 and all of them show only the reflections of the desired compounds^12^,^13^, with no lead (II) halides detected.

**Figure 2:**
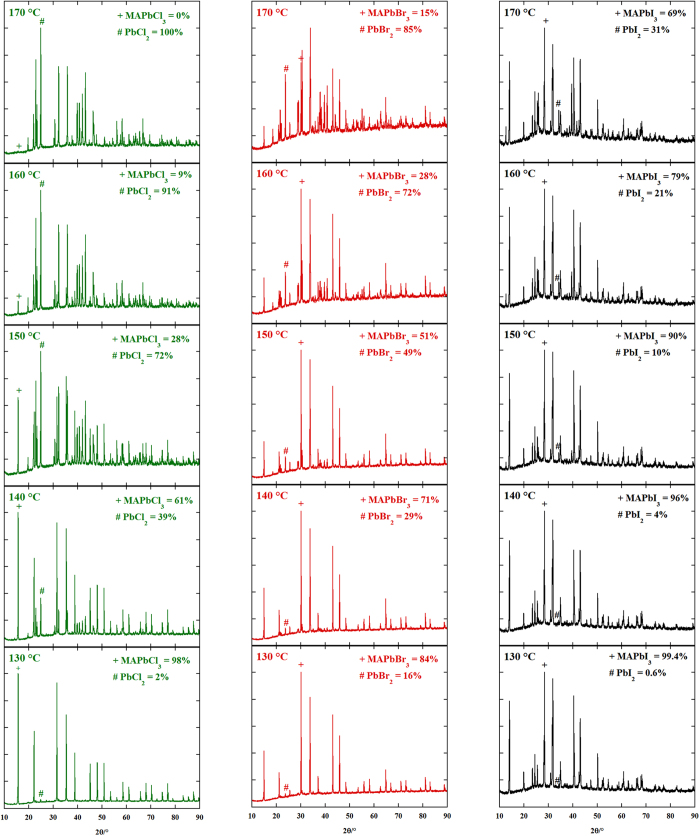
X-ray diffraction patterns of the MAPbX_3_ compounds after each isotherm in the non-ambient reactor chamber. The temperature of the isotherm after which the pattern was taken, as well relative weight percentages of the phases are reported inside each sub-panel. The symbols “+” and “#” are used as guide to the eye to appreciate two characteristic reflections (one for each phase) and their relative variation after each thermal treatment.

The compounds were then inserted into the non-ambient reactor chamber and underwent a thermal treatment under helium atmosphere from 130 °C to 170 °C with isotherms every 10 °C, each one lasting 10 hours. After each isotherm the sample was quickly cooled to 25 °C and a diffraction pattern was taken. The temperature range was chosen in order to have detectable changes of the samples within the isotherm duration. The thermal profile used in the experiments is shown in Figure S1 of the Supplementary Information. The diffraction patterns after each isotherm for each compound are given in Fig. 2, while a magnification of the same, together with the reflections of the corresponding MAPbX_3_ and PbX_2_ are shown in Figures S2–S4 of the Supplementary Information. The phase identification analysis performed on the patterns revealed that the only solid decomposition product is the corresponding lead (II) halide for all the compounds under investigation. No trace of solid methylammonium halides has been found. This fact, together with the results coming from the Knudsen effusion-mass spectrometry (KEMS) and Knudsen effusion mass loss experiments (KEML), demonstrate that the decomposition reactions occur in all cases with the loss of gaseous methylamine (MA) and the corresponding hydrogen halide HX according to the reaction:


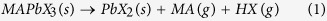


and not by phase separation of solid lead (II) halides and methylammonium halides, a possibility that had to be taken into account considering that all the methylammonium halides under consideration have melting points over 200 °C.

**Figure 3:**
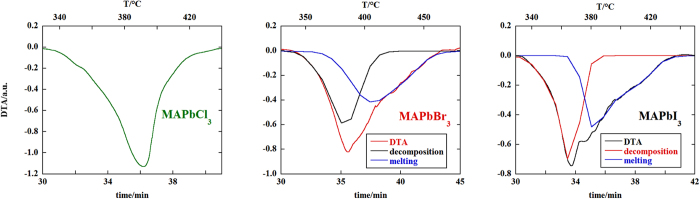
Endothermic DTA peaks for the decomposition reaction of MAPbX_3_ compounds. In the case of MAPbBr_3_ and MAPbI_3_ the endothermic peak of the decomposition event convolutes with the endothermic peak of the melting process of the corresponding lead (II) halide, so the contribution of each process was separated by a peak fitting procedure (sum of gaussians). The “shape index” of each decomposition peak has been used to obtain the order of reaction.

After phase identification, the quantitative phase analysis was performed by applying the Rietveld method on the diffractograms. The relative weight percentages of the crystalline phases are reported in the diffraction patterns shown in Fig. 2. The order of reaction for the decomposition reactions has been determined by differential thermal analysis (DTA) of the compounds using the “shape index” of the endothermic decomposition peak in the thermograms^14^. The DTA peaks for decomposition of the compounds are shown in Fig. 3. For MAPbBr_3_ and MAPbI_3_ the peaks due to the melting processes of the corresponding lead (II) halides partially overlap with those of the decomposition, so a peak fitting procedure with a sum of gaussian functions was applied to separate the contributions of each process to the resulting DTA peak. All the compounds decompose according to a first order kinetics.

Considering then a first order kinetics for the decomposition of the MAPbX_3_ compounds, and using the integrated form of the rate equation


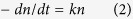


i.e.:


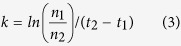


the values of the kinetic constant *k* can be obtained at each of the temperatures of the isotherms used in the experiments; *n* is the number of moles of reactant, *t* is the time, and the number 1 and 2 indicate initial and final state, respectively.

**Figure 4:**
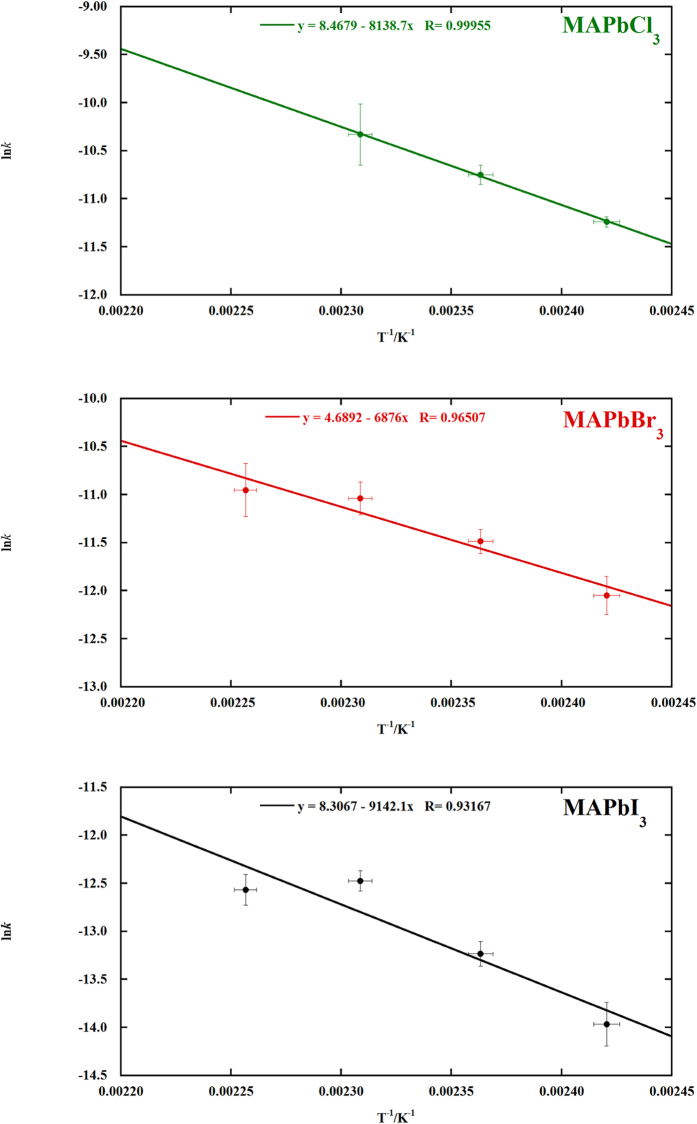
Plot of the values of ln *k* vs. the absolute temperature. From the slope of each linear fit, the values of the apparent activation energies for the decomposition reaction of each MAPbX_3_ compound were calculated and from the equation of the lines the *k* and consequently the 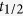 values at 90 °C were estimated.

By plotting the values of ln *k* vs the inverse of the absolute temperature, and fitting them with a straight line, the apparent activation energies for the decomposition reactions can be obtained by the slope of the line, considering the Arrhenius equation:


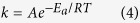


where *A* is the prefactor, *E*_*a*_ the apparent activation energy, *R* the gas constant and *T* the absolute temperature. These plots are shown in Fig. 4. In all the cases the point at 130 °C was excluded because of the excessive uncertainty on the value of ln *k*. In the case of MAPbCl_3_ the value of ln *k* at 170 °C cannot be calculated because the decomposition of MAPbCl_3_ was complete and so equation (3) cannot be applied. The obtained values of the apparent activation energies for the decomposition reactions are, considering the uncertainties, practically equal, being 68 ± 2 kJ/mol, 60 ± 10 kJ/mol and 80 ± 20 kJ/mol for MAPbCl_3_, MAPbBr_3_ and MAPbI_3_, respectively.

For an estimation of the lifetime of the compounds under severe operative conditions of the devices, a value of *k* at 90 °C (which is a temperature that solar cells can reach under harsh conditions^15^) can be extrapolated, and from the value of *k*, the half life


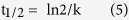


of the compounds can be calculated. The estimated half-lives are 9, 12 and 170 days for MAPbCl_3_, MAPbBr_3_ and MAPbI_3_, respectively. These values, though being simply rough estimations, pose a significant question about the possibility of using these compounds for the realization of stable solar cells, not considering all the problems related to other causes of degradation.

### Thermodynamic stability: Knudsen effusion mass spectrometry and Knudsen effusion mass loss

Evaporation studies were performed with two different techniques based on molecular effusion: KEMS (Knudsen Effusion Mass Spectrometry) and KEML (Knudsen Effusion Mass Loss). Details of the experimental apparatus and techniques are given in the Methods section.

With KEMS the nature and abundance of the effusing molecular species can be determined from the mass-spectrometrically detected ions and their measured intensities. In all three of the MAPbX_3_ compounds here under study mass spectra were consistent with the aforementioned decomposition process (1), leading to the formation in the gas phase of methylamine and the corresponding hydrogen halide. Mass spectra were almost coincident with the reference spectra of such compounds. The relative intensities of methylamine and hydrogen halide ions varied depending on the system, as expected. Indeed, the fundamental equation correlating the individual molecular fluxes, 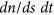, and the corresponding molecular densities *n*′ inside the cell is:


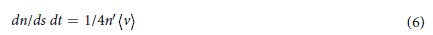


where 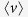
is the average molecular speed. Fluxes and pressure are therefore correlated through the factor 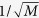, where *M* is the molecular mass. In order to maintain the stoichiometry of reaction (1), equal escaping fluxes of methylamine and HX are required, therefore the partial pressure ratios must satisfy the relation:


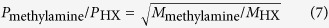


Ratios are thus expected to be 0.93, 0.62 and 0.49 for MAPbCl_3_, MAPbBr_3_ and MAPbI_3_, respectively. In our experiments the corresponding measured average ratios were found to be 1.01 ± 0.46, 0.56 ± 0.24 and 0.24 ± 0.10. While these ratios, even if largely scattered, are consistent with the decomposition stoichiometry of reaction (1) for MAPbCl_3_, MAPbBr_3_, a ratio smaller than expected was observed for MAPbI_3_. However, it should be noted that (see the Methods section), two species-dependent parameters affect the partial pressures derived from ion intensities: the electron impact cross section and the multiplier gain. Uncertainties in the estimation of these parameters would in turn affect the measured ratio. In this regard, we note that the deviation from the theoretical ratio increases with the difference between the masses of methylamine and HX, suggesting an inaccuracy in the estimation of the multiplier gain, which is usually assumed to be proportional to the reciprocal square root of the molecular mass. In conclusion, the occurrence of decomposition reactions as per equation (1) can be confidently considered to be consistent with the KEMS results. Moreover, as already noted, the X-ray diffraction analysis of the vaporization residues did not show any phase other than MAPbX_3_ compounds and the corresponding lead (II) halides, so supporting these findings. Note that this decomposition behavior is the same observed for pure methylammonium chloride^16^.

The discussion reported above on the partial pressure ratios measured with KEMS and the possible uncertainties in their determination paves the way for three alternative derivations of the equilibrium constant of reactions (1). Indeed one can chose to use either of the two partial pressures (methylamine and hydrogen halide) to evaluate the equilibrium constant, by imposing that the other one do obey the ratio consistent with equation (1). For example, assuming the measured partial pressure of methylamine as more reliable, the equilibrium constant *K*_*p*_ can be evaluated as:


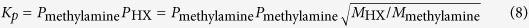


As a third alternative, partial pressures measured for both species can be directly used in the *K*_*p*_ evaluation. Considering the various uncertainties involved, such as ion current measurement, fragmentation processes, as well as cross section and multiplier gain estimations, we deemed the procedure based on the hydrogen halide partial pressure measurement as the most reliable. The methylamine and hydrogen halide partial pressures evaluated with this method are reported in Table 1 of the supplementary information for all the MAPbX_3_ compounds here under study. Results so derived will be detailed below, whereas the other two procedures will be used as an aid in estimating the overall reliability of the results.

In the experiments with the KEML technique (see the Method section), the observable is the overall rate of mass loss of the Knudsen effusion cell. In order to relate this quantity with the total pressure inside the cell the occurrence of the decomposition reaction (1) was assumed, consistently with mass spectrometric findings. The total pressures collected are reported in Table 2 of the supplementary information. In turn, the equilibrium constant of this very same reaction can be related to the total pressure taking into account, as seen before, the relation between the partial pressures of methylamine and HX and the corresponding effusing fluxes:


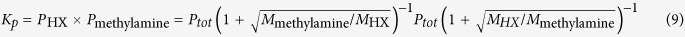


The resulting factors multiplicative of 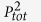
are 0.250, 0.236 and 0.221 for the decomposition reaction of the chloride, bromide and iodide compound, respectively. It is also to be noted that, in the case of the KEML technique, experiments have been performed with different size of the effusion orifice (1 mm and 3 mm diameter) in search of the occurrence of any kinetically hindered evaporation, as it is sometimes reported for decomposition reactions^17^,^18^. Within the experimental uncertainties, however, no difference was observed in the total pressures measured with different orifices.

All the *K*_*p*_ data collected for reaction (1) experiments were analyzed with the two thermodynamic processing procedures known as second- and third-law methods, detailed in the Methods section. The main results of this analysis are listed in Table 1, where we report the enthalpy change for process (1) derived by applying the two methods to *K*_*p*_ values obtained by KEML and by KEMS (in the latter case, by using the HX pressures to evaluate *K*_*p*_). Enthalpy change derived from the second-law analysis are reported both at the mean temperature of the experiments and to the reference temperature of 0 K.

On looking at the results of Table 1 it is apparent, for all the three systems, an excellent agreement between the third-law enthalpies of reaction derived by the two techniques. On comparing the second- and third-law results a more complex pattern comes out. While a rather good agreement was obtained for KEMS results in the case of MAPbCl_3_, MAPbBr_3_ compounds, in the other cases the second law enthalpies are larger than the third-law ones by 7 to 18 kJ/mol. The quality of the original data is therefore to be evaluated as somewhat scattered. In addition, the second-law KEMS results were significantly dependent on which method, among the three mentioned above, is used to evaluate *K*_*p*_, the scatter of the resulting enthalpies of reaction being in the order of 10–15 kJ/mol. The corresponding scatter of the third law values, on the contrary, was found to be of the same order of the small statistical errors reported in Table 1.

Generally, the third law treatment of data is considered to be of superior quality if it is based, as it is here the case, on reliable free energy function values. The excellent agreement of the results obtained with the two independent experimental techniques KEMS and KEML leads us to take into account only the third-law results in proposing the final average values for the enthalpies of the reactions (1):


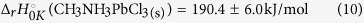



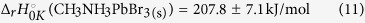



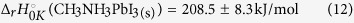


The probable errors associated to the proposed values have been evaluated as half the maximum spread resulting both from the KEMS second-law values derived from the three different procedures used to calculate *K*_*p*_ from pressure data (see above) and the difference between the third-law enthalpy and the corresponding second law value.

The enthalpy change of reactions (1) allows one to derive the formation enthalpy of the corresponding perovskite phases at the usual reference temperature of 298 K. To this end, the formation enthalpies of gaseous hydrogen halides and solid lead halides were taken from the IVTANTHERMO database^19^, whereas the value for methylamine was retrieved from the compilation of ref. 17. The resulting values of 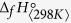 were: −688.3 ± 7.8 kJ/mol, −567.5 ± 8.7 kJ/mol, and −403.6 ± 9.7 kJ/mol for MAPbCl_3_, MAPbBr_3_ and MAPbI_3_, respectively. Note that the decreasing trend of 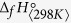 absolute values is essentially due to the trend observed for the corresponding lead (II) halide and hydrogen halide species.

The above derived enthalpies of decomposition enable us to evaluate the corresponding standard Gibbs energy changes as a function of temperature, useful for equilibrium calculations and thermodynamic simulations within a temperature range reasonably close to that covered in our experiments. To this end, the standard enthalpies and entropies of decomposition at the average experimental temperatures (see Table 1) were evaluated with the heat content and free energy functions. The following expressions are finally obtained:


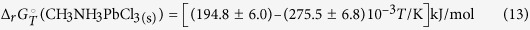



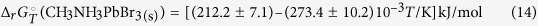



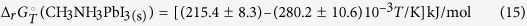


**Figure 5:**
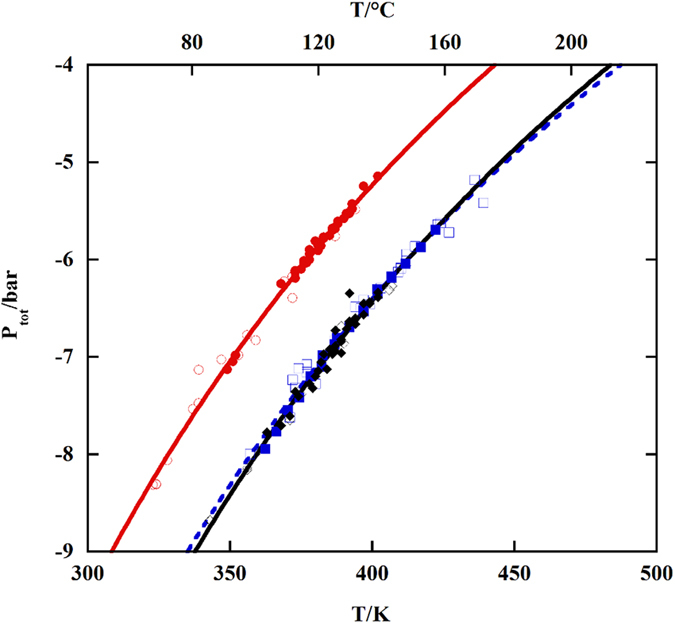
Total decomposition pressure (in bar) of the perovskite phases under study as a function of the temperature. Circles, squares and rhombus for MAPbCl_3_, MAPbBr_3_ and MAPbI_3_, respectively. Open symbols refer to the KEMS experiments, closed ones to KEML.

In conclusion, our results suggest a more pronounced thermodynamic tendency of the MAPbCl_3_ towards decomposition compared to MAPbBr_3_ and MAPbI_3_. In order to facilitate an easy appreciation, from a practical point of view, of the stability of the studied perovskite phases, the total pressure dependence on the temperature is displayed in Fig. 5.

It is worth noting that, by comparing the kinetic and thermodynamic data presented above, MAPbCl_3_ results to be the most unstable compound both kinetically and thermodynamically. With regard to MAPbBr_3_ and MAPbI_3_, while these two compounds have practically identical decomposition pressures in the explored temperature range (see Fig. 5), MAPbI_3_ is kinetically much more stable, as shown in Fig. 4.

In summary, the studies of the MAPbX_3_ compounds presented in this work clarify experimentally for the first time their decomposition path and highlight their limited thermal and thermodynamic stabilities and these are additional intrinsic problems that should be addressed in order to exploit their potential as photovoltaic materials in real life devices.

## Methods

### Materials preparation

Lead (II) acetate trihydrate (99.0–103%), lead (II) chloride (reagent grade, 99%), hydrobromic acid 47% in water, hydroiodic acid 57% in water (stabilized with 1.5% hypophosphorous acid), methylamine 40% in water were purchased from Alfa Aesar. Hydrochloric acid 37% in water was purchased from Sigma Aldrich. The compounds were synthesized as powders according to literature procedure^20^. In the case of MAPbCl_3_ a quantity of 37% HCl double with respect to literature procedure was necessary to dissolve the solid PbCl_2_. All the syntheses gave crystalline precipitates that were collected on a Büchner funnel under suction. In the case of MAPbI_3_ the filtration was performed while the solution was still hot (T > 45–50 °C) to avoid the formation of (MA)_4_PbI_6_∙2H_2_O^21^. After filtration, the solids were left under suction for at least 30 min to let them dry. MAPbCl_3_ and MAPbBr_3_ were then washed with acetone to remove the last traces of the mother solutions and left under suction for additional 30 min. The washing with acetone was not possible in the case of MAPbI_3_, which immediately decomposes in contact with the solvent, probably because of the traces of water present in the same. So after drying under suction, it was purified by keeping it in vacuum at 100 °C overnight.

### X-ray diffraction and non-ambient X-ray diffraction

X-ray diffraction patterns on the as-synthesized samples were performed by using a Panalytical X’Pert Pro MPD diffractometer (Cu Kα radiation, λ = 1.54184 Å) equipped with an ultra-fast X’Celerator RTMS detector. The angular resolution (in 2θ) was 0.001°. A 0.04 rad soller slit, a 1° divergence slit and a 20 mm mask have been used on the incident beam path, while a 6.6 mm anti-scatter slit, a Ni Kβ filter and a 0.04 rad collimator have been used on the diffracted beam path.

For the non-ambient X-ray diffraction measurements, the diffractometer has been equipped with an Anton Paar XRK 900 reactor chamber with a factory calibrated automatic stage mover, using a Macor glass-ceramic sample holder. The position of the sample holder was calibrated at room temperature using the (104) reflection of powdered corundum. The experiments have been performed at atmospheric pressure under a protective flow of He gas (20 cm^3^/min @ STP, purity 99.999%). The optics used for the non-ambient measurements were the same as above with the exception of incident beam path mask (15 mm instead of 20 mm).

All the scans were performed in the angular range 10–90° (in 2θ) with a scan time of 1 hour.

The Rietveld analyses of the diffractograms taken in non-ambient conditions were performed by using the MAUD software package^22^. The necessary cif (crystallographic information file) files for the MAPbX_3_ compounds were created from the literature data^12^,^23^, while for the lead (II) halides they were retrieved from the FIZ/NIST ICSD (Inorganic Crystal Structure Database) FindIt database^24^.

### Differential Thermal Analysis (DTA)

DTA measurements on the MAPbX_3_ compounds were performed using a Netzsch STA 409 PC Luxx thermal analyzer. The DTA sensor was calibrated against the melting points of In, Sn, Zn, Al, Ag, Au and Ni at least 99.9% pure. The measurements were performed in sintered alumina crucibles under flowing Ar atmosphere (85 cm^3^/min @ STP, purity ≥ 99.9995%) with a scan rate of 10 K/min.

### Knudsen Effusion Mass Spectrometry (KEMS)

The features of the Knudsen effusion mass spectrometry (KEMS) technique are well summarized in ref. 25 and references cited therein. The apparatus employed is a single focusing 90° magnetic sector mass spectrometer, originally by Patco, equipped with a Knudsen molecular source^26^. Graphite effusion cells with 1 mm diameter effusion holes, inserted in an outer molybdenum crucible, were used. The molecular source assembly is surrounded by a spiral-shaped tungsten as heating element and several tantalum shields. The temperature of the cell was measured with a Pt-Pt/Rh 10% thermocouple inserted in the bottom of the molybdenum container. Ionization of the vapours originated by the Knudsen molecular source was accomplished by electron impact with an electron emission current generally regulated at 1.0 mA. A secondary electron multiplier was used as a detector. The basic experimental data are the ion intensities, 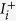, recorded as a function of the temperature of the molecular source. These can be converted into partial pressures of the corresponding neutral species in the Knudsen cell through the relation^25^:


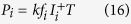


where *k* is the instrument sensitivity constant and the factor:


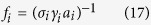


specific to each ion *i*, includes the electron impact ionization cross section 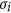, the multiplier gain 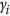, and the isotopic abundance *a*_*i*_. The instrument sensitivity constant has been evaluated, in the course of this study, with separate experiments of vaporization of pure zinc, whose vapor pressure is well known^19^. The ionization cross section of methylamine (5.60 Å^2^) was taken from ref. 27, those for HCl (3.76 Å^2^), HBr (4.65 Å^2^) and HI (6.47 Å^2^) from ref. 28. The multiplier gains were assumed to be proportional to the inverse square of the molecular ion mass, as usual in KEMS^25^.

### Knudsen Effusion Mass Loss (KEML)

A Ugine-Eyraud Model B60 Setaram thermobalance was used for the KEML measurements. A graphite resistor is the heating element of a quartz tube containing the Knudsen cell. The effusion source, whose mass is monitored, was specifically modified in our laboratory^29^ in order to allow an optimal temperature measurement and to maximize the uniformity of the sample temperature. Briefly, both the Knudsen cell and a Pt100 platinum resistance thermometer are inserted into a capped copper cylinder; so that the temperature of the molecular source is directly measured instead of the usual “dummy” cell placed in the isothermal section of the furnace. Effusion cells made of pyrophyllite were used, with effusion hole diameters of 1 and 3 mm. The vapor pressures of the sample were obtained by the usual Knudsen equation:


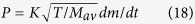


where *T* is the temperature, *K* a constant depending on the geometrical characteristics of the effusion hole, *dm/dt* the rate of mass loss. *M*_*av*_ is the average molar mass of the effusing vapor:


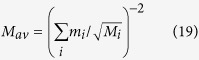


where *m*_*i*_ are the weight fractions of the various vapor species in the effusate and *M*_i_ the respective molecular weights.

### Thermodynamic analysis of *K*_*p*_ data

Equilibrium data have been analyzed by the so-called second- and third-law methods of analysis^25^. With the second-law method the determination of the enthalpy change at the average temperature of the experiment, 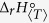, is performed by a least square analysis of a van’t Hoff plot, ln *K*_p_ vs. 1/*T*, where *K*_*p*_ is the equilibrium constant. The corresponding enthalpy change at the reference temperature, in our case 0 K, 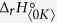, can be calculated through the use of the heat content functions, 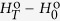 (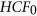) of reactants and products. A single 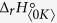 value is obtained from the entire set of data points. The third-law procedure, based on the relation:


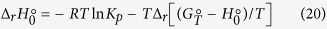


where 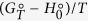 is the Gibbs energy function 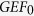, provides a value of 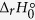 for each experimental point. The advantages and shortcomings of these two independent methods of analysis of primary experimental data are reported in ref. 25. In short, although the third law analysis, unlike the second-law, requires the more demanding knowledge of the absolute values of partial pressures, third-law results are considered to be superior and to be preferred when thermal functions are sufficiently well established, because they are less sensitive to random errors.

### Auxiliary thermodynamic functions

Free energy functions of gaseous hydrogen halides and solid lead halides were retrieved from the IVTANTHERMO database^19^, whereas those of methylamine were taken from ref. 30. The heat capacity 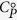 of the MAPbX_3_ compounds have been measured in ref. 31 up to 300 K for MAPbCl_3_ and MAPbBr_3_, and up to 360 K for MAPbI_3_. The same authors derived, in the same temperature range, the thermodynamic functions 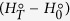, 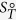, 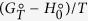. These thermodynamic functions have been here evaluated up to 450 K in order to cover the range of temperatures of our experiments. To this end, heat capacity data were extrapolated using the Einstein and Debye temperatures provided by the same authors. The resulting values are reported in the Table 3 of the Supplementary information.

## Additional Information

**How to cite this article**: Brunetti, B. *et al.* On the Thermal and Thermodynamic (In)Stability of Methylammonium Lead Halide Perovskites. *Sci. Rep.* **6**, 31896; doi: 10.1038/srep31896 (2016).

## Supplementary Material

Supplementary Information

## Figures and Tables

**Table 1:** Summary of the KEMS and KEML experiments and enthalpy changes for the decomposition reactions derived thereafter.

	CH_3_NH_3_PbCl_3(s)_ = PbCl_2(s)_+CH_3_NH_2(g)_ + HCl_(g)_		CH_3_NH_3_PbBr_3(s)_ = PbBr_2(s)_+CH_3_NH_2(g)_+HBr_(g)_		CH_3_NH_3_PbI_3(s)_ = PbI_2(s)_+CH_3_NH_2(g)_+HI_(g)_	
	KEMS	KEML	KEMS	KEML	KEMS	KEML
n of data	18	40	25	20	14	31
*T*_*av*_/K	353	381	398	391	367	386
second-law 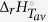 /kJ mol^−1^	190.8 ± 6.6	197.6 ± 2.4	196.8 ± 5.8	217.5 ± 3.1	209.2 ± 4.4	219.3 ± 7.1
second-law 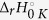 /kJ mol^−1^	195.5 ± 6.6	202.5 ± 2.4	201.2 ± 5.8	221.8 ± 3.1	216.1 ± 4.4	226.4 ± 7.1
third-law 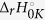 /kJ mol^−1^	190.8 ± 1.7	190.0 ± 2.4	207.4 ± 1.6	208.1 ± 0.8	208.8 ± 1.1	208.0 ± 1.0
proposed 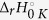 /kJ mol^−1^	190.4 ± 8.0		207.8 ± 10.5		208.4 ± 10.9	

Uncertainties on second- and third-law values are standard deviations, whereas the final error associated to the proposed value is estimated as described in the text.
