# Corrigendum: On the Thermal and Thermodynamic (In)Stability of Methylammonium Lead Halide Perovskites

**DOI:** 10.1038/srep46867

**Published:** 2017-07-27

**Authors:** Bruno Brunetti, Carmen Cavallo, Andrea Ciccioli, Guido Gigli, Alessandro Latini

Scientific Reports 6: Article number: 31896; 10.1038/srep31896 published online: 07 22 2016; updated: 07 27 2017.

This Article contains errors. In the ‘Thermal Stability: Non-Ambient X-ray Diffraction’ section, under subheading ‘Thermodynamic stability: Knudsen effusion mass spectrometry and Knudsen effusion mass loss’,

“The resulting values of 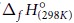 were: −688.3 ± 7.8 kJ/mol, −567.5 ± 8.7 kJ/mol, and −403.6 ± 9.7 kJ/mol for MAPbCl_3_, MAPbBr_3_ and MAPbI_3_, respectively”.

should read:

“The resulting values of 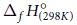 were: −660.5 ± 7.8 kJ/mol, −539.6 ± 8.7 kJ/mol, and −375.7 ± 9.7 kJ/mol for MAPbCl_3_, MAPbBr_3_ and MAPbI_3_, respectively”.

In Equation (13),

“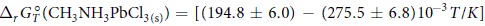 kJ/mol”

should read:

“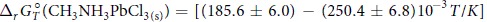 kJ/mol”

In Equation (14),

“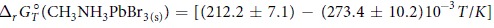 kJ/mol”

should read:

“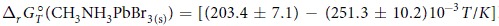 kJ/mol”

In Equation (15),

“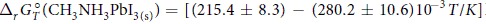 kJ/mol”

should read:

“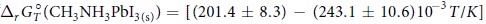 kJ/mol”

