# Supplementary material for: On the Thermal and Thermodynamic (In)Stability of Methylammonium Lead Halide Perovskites
Source: Sci Rep. 2016 Aug 22;6:31896. doi: 10.1038/srep31896 (PMC4992962; doi:10.1038/srep31896)
Supplement: Supplementary Information [file srep31896-s1.pdf]

Supplementary Information to the Manuscript

On the Thermal and Thermodynamic (In)Stability of Methylammonium Lead Halide Perovskites

Bruno Brunetti, Carmen Cavallo, Andrea Ciccioli*, Guido Gigli, Alessandro Latini*

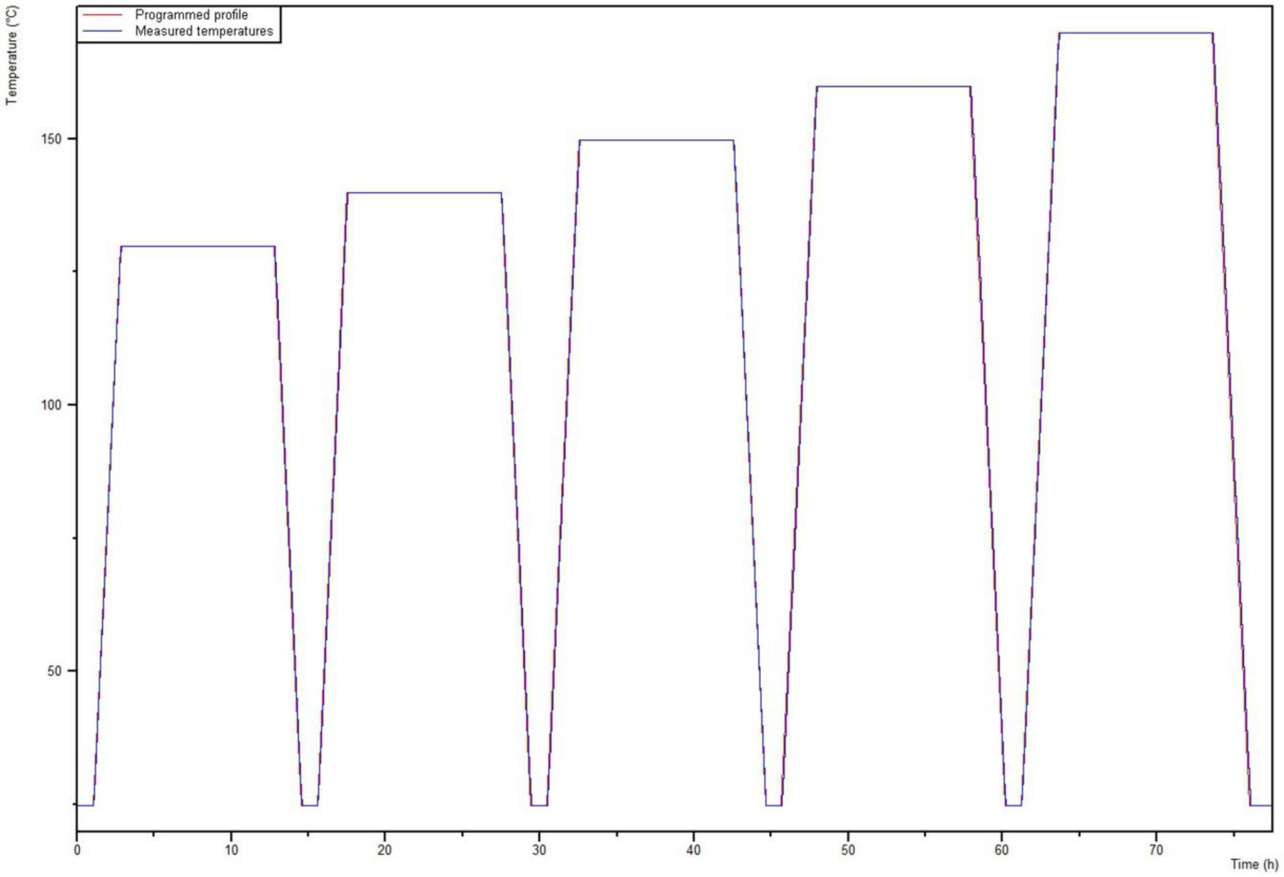

Figure S1. Thermal profile used during the non-ambient X-ray diffraction measurements.

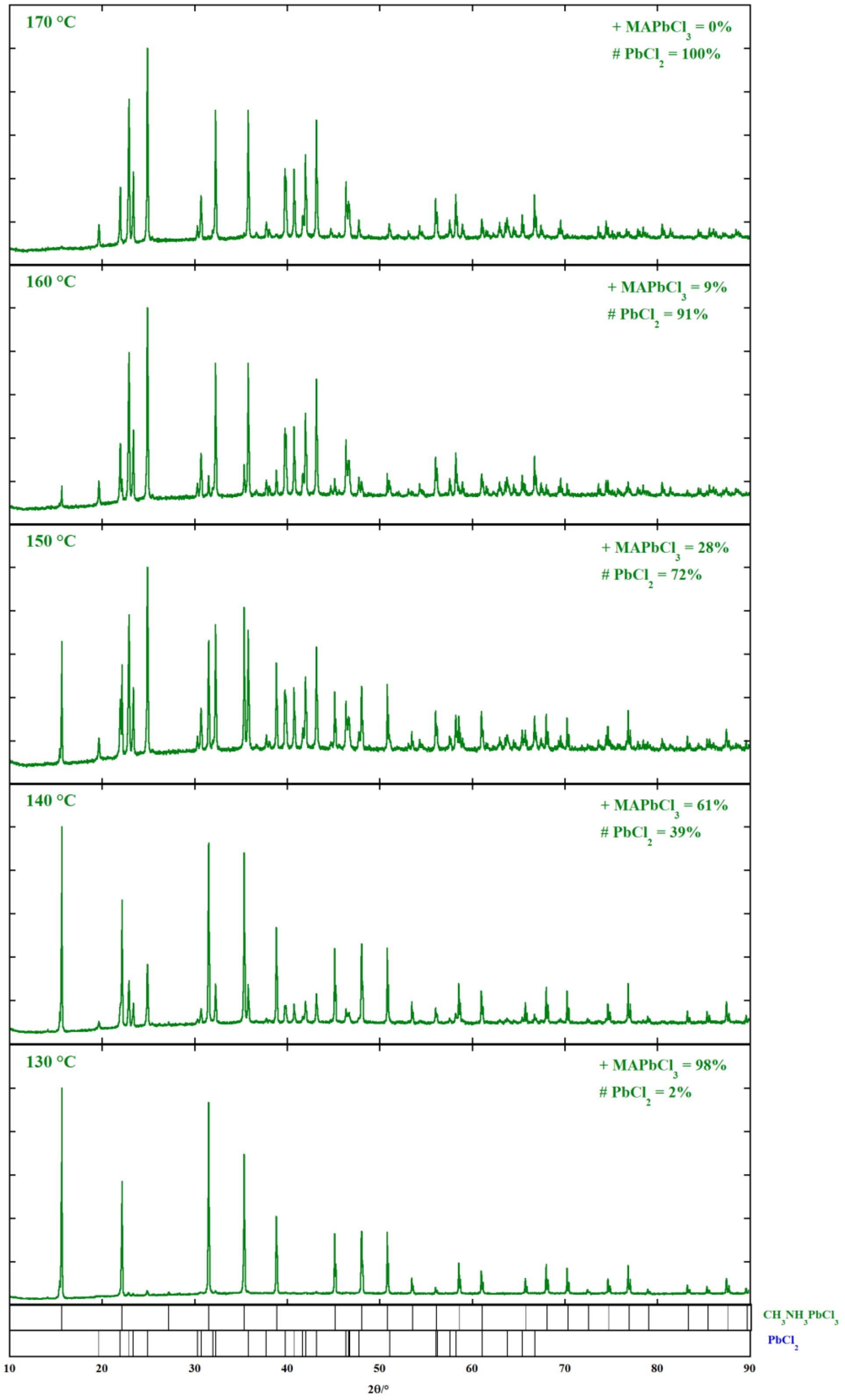

Figure S2. Magnification of the diffraction patterns of $MAPbCl_3$ taken after each isotherm compared with the reference reflections of $MAPbCl_3$ and $PbCl_2$.

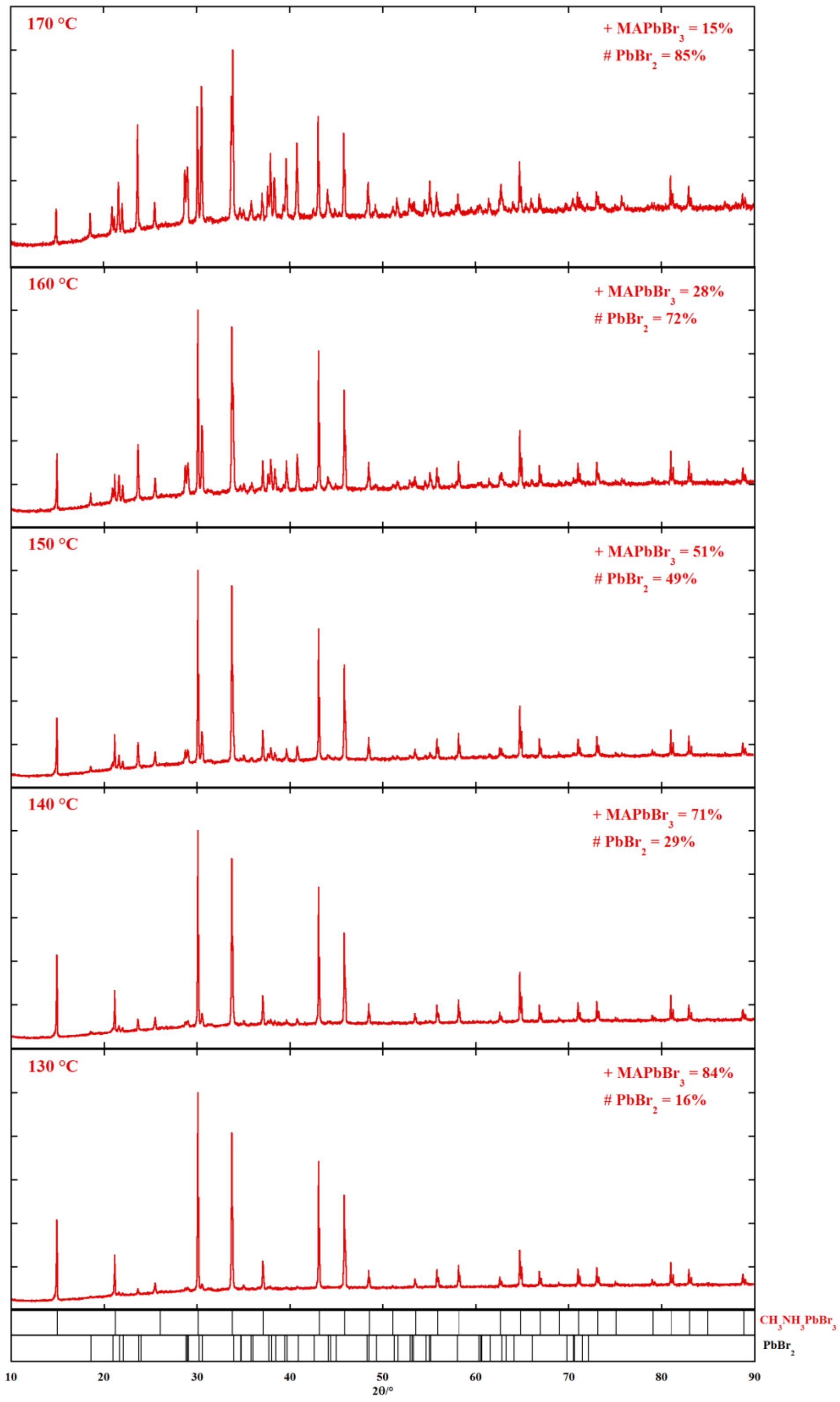

Figure S3. Magnification of the diffraction patterns of $MAPbBr_3$ taken after each isotherm compared with the reference reflections of $MAPbBr_3$ and $PbBr_2$.

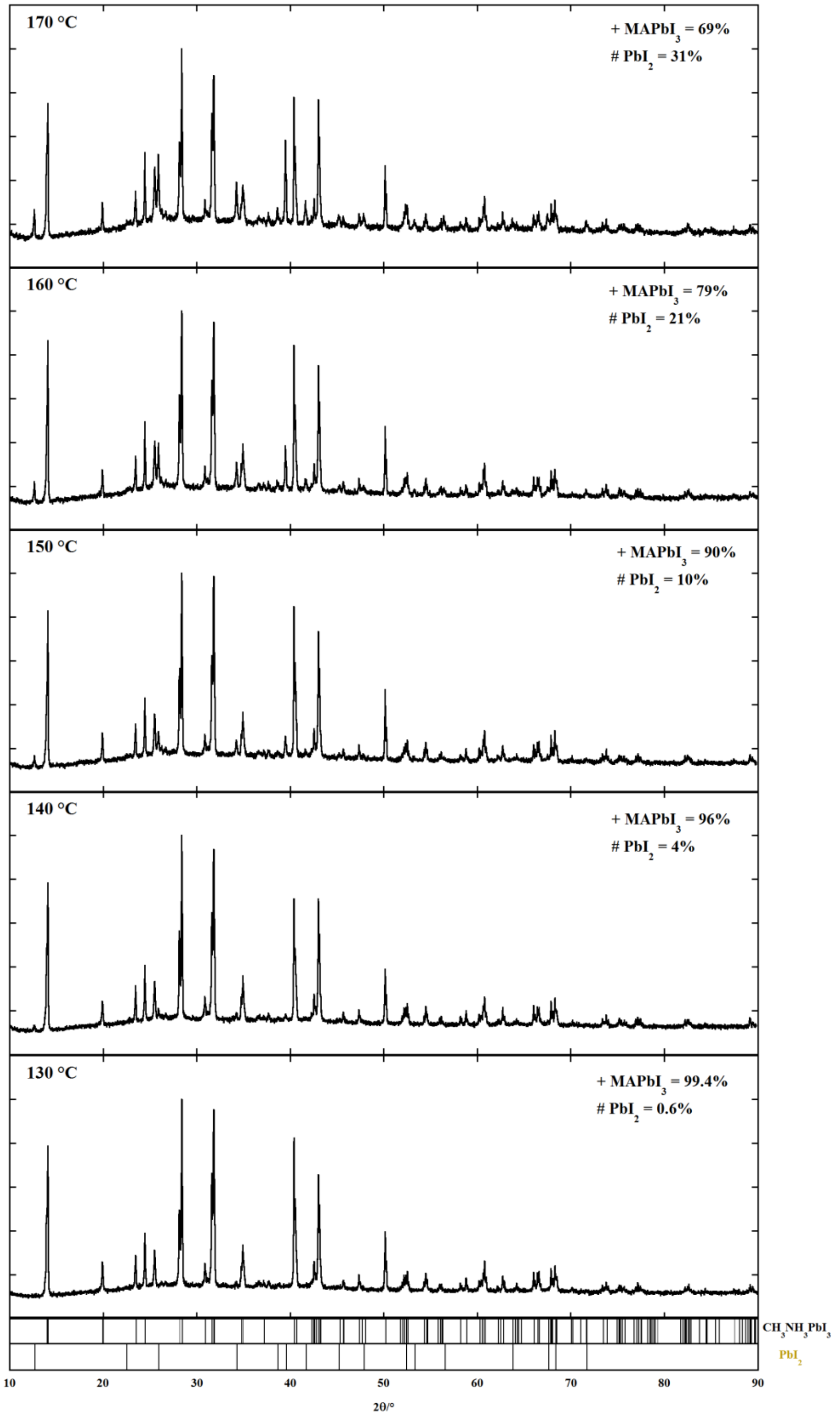

Figure S4. Magnification of the diffraction patterns of $MAPbI_3$ taken after each isotherm compared with the reference reflections of $MAPbI_3$ and PbI_2.

Table 1. Partial pressures (in bar) derived from KEMS measurements.

T/K	P(HCl)	P(MetAm)	T/K	P(HBr)	P(MetAm)	T/K	P(HI)	P(MetAm)
353	5.40E-08	2.35E-08	371	1.48E-08	1.32E-08	360	7.24E-09	1.20E-09
324	2.54E-09	9.42E-10	396	1.97E-07	4.35E-08	343	1.41E-09	1.94E-10
337	1.51E-08	4.32E-09	394	2.02E-07	4.18E-08	371	1.51E-08	2.30E-09
369	3.11E-07	9.08E-08	409	4.60E-07	9.49E-08			
			410	5.00E-07	9.78E-08			
			377	4.33E-08	8.99E-09			
						389	1.39E-07	2.79E-08
339	3.82E-08	6.67E-08	372	3.59E-08	3.07E-08	406	3.60E-07	7.95E-08
372	2.09E-07	3.35E-07	377	5.20E-08	4.15E-08	406	3.25E-07	7.95E-08
387	9.02E-07	9.20E-07	397	2.38E-07	1.93E-07	373	2.53E-08	5.24E-09
324	2.56E-09	3.90E-09	410	5.09E-07	4.18E-07	356	4.62E-09	1.14E-09
			412	5.97E-07	4.65E-07			
356	8.73E-08	9.26E-08	427	1.18E-06	6.58E-07	343	1.40E-09	4.08E-10
372	3.47E-07	3.49E-07	380	3.28E-08	3.24E-08	342	1.16E-09	3.19E-10
387	1.12E-06	1.09E-06				375	2.80E-08	7.32E-09
323	2.52E-09	4.48E-09	412	7.06E-07	5.66E-07	390	9.20E-08	2.34E-08
			415	8.48E-07	6.46E-07	390	1.06E-07	2.34E-08
339	1.74E-08	1.78E-08	399	2.14E-07	1.36E-07	356	4.84E-09	1.22E-09
376	4.85E-07	4.64E-07				356	4.08E-09	1.22E-09
394	1.70E-06	1.60E-06						
328	4.51E-09	6.36E-09	387	9.18E-08	4.26E-08	326	1.41E-10	5.99E-11
			403	3.13E-07	1.36E-07	326	1.56E-10	9.11E-11
347	4.89E-08	4.74E-08	403	3.13E-07	1.39E-07	374	2.98E-08	7.00E-09
359	7.71E-08	8.31E-08				374	3.14E-08	7.00E-09
			424	1.47E-06	6.56E-07	407	3.63E-07	7.42E-08
			424	1.47E-06	7.38E-07			
			424	1.42E-06	6.70E-07			
			424	1.42E-06	6.01E-07			
			373	2.98E-08	1.52E-08			
			357	6.19E-09	3.33E-09			
			374	4.74E-08	2.86E-08			
			436	4.07E-06	1.72E-06			
			439	2.37E-06	1.07E-06			

Table 2. Total pressures measured in KEML experiments. For each system, data with effusion holes of 1 and 3 mm in diameter are reported in sequence.

Chloride		Bromide		Iodide	
T/K	P_{tot}/bar	T/K	P_{tot}/bar	T/K	P_{tot}/bar
402	7.16E-06	387	1.35E-07	387	1.15E-07
397	5.68E-06	382	1.04E-07	392	2.20E-07
393	3.75E-06	388	1.58E-07	397	3.56E-07
388	2.49E-06	392	2.11E-07	402	4.60E-07
383	1.66E-06	397	3.03E-07	399	3.55E-07
378	1.14E-06	402	4.97E-07	394	2.51E-07
373	7.59E-07	407	6.76E-07	389	1.52E-07
368	5.66E-07	412	9.13E-07	385	1.20E-07
381	1.24E-06	417	1.35E-06	380	6.37E-08
386	2.09E-06	422	2.03E-06	374	3.93E-08
391	2.92E-06	392	2.02E-07	382	8.85E-08
393	3.75E-06	397	2.94E-07	387	1.31E-07
383	1.70E-06	402	4.47E-07	392	2.33E-07
378	1.26E-06	407	6.51E-07	397	2.72E-07
373	7.66E-07			402	4.10E-07
376	9.07E-07	382	7.94E-08	399	3.66E-07
381	1.40E-06	378	6.33E-08	394	2.18E-07
386	2.06E-06	374	3.82E-08	389	1.44E-07
391	2.98E-06	370	2.86E-08		
393	3.38E-06	366	1.72E-08	392	4.52E-07
388	2.35E-06	362	1.14E-08	387	1.87E-07
380	1.55E-06			383	1.07E-07
378	1.00E-06			373	4.42E-08
373	7.42E-07			363	1.68E-08
376	9.75E-07			391	1.94E-07
381	1.41E-06			386	1.07E-07
386	2.01E-06			381	7.20E-08
391	2.89E-06			378	5.31E-08
393	3.32E-06			371	2.47E-08
390	2.63E-06			368	1.98E-08
385	1.76E-06			389	1.10E-07
392	2.98E-06			384	7.52E-08
387	2.07E-06			379	4.80E-08
382	1.39E-06				
377	9.28E-07				
373	6.48E-07				
375	7.99E-07				
352	1.05E-07				
351	9.00E-08				
349	7.48E-08				

Table 3. Thermodynamic functions of the perovskite phases extrapolated from values reported in Ref. 28.

T/K	C_p° / R	$\left(H_T^\circ - H_0^\circ\right)/ RT$	S_T° / R	$\left(G_T^\circ - H_0^\circ\right)/ RT$
**$CH_3NH_3PbCl_{3(s)}$**				
310	20.49	17.00	38.49	-21.49
320	20.51	17.11	39.14	-22.03
330	20.53	17.21	39.77	-22.55
340	20.56	17.31	40.39	-23.07
350	20.57	17.40	40.98	-23.57
360	20.59	17.49	41.56	-24.06
370	20.61	17.57	42.13	-24.54
380	20.62	17.65	42.68	-25.06
390	20.64	17.73	43.21	-25.47
400	20.65	17.80	43.74	-25.92
410	20.66	17.87	44.25	-26.36
420	20.67	17.94	44.74	-26.80
430	20.68	18.00	45.23	-27.22
440	20.69	18.06	45.71	-27.63
450	20.70	18.12	46.17	-28.04
**$CH_3NH_3PbBr_{3(s)}$**				
310	20.52	17.63	42.81	-25.19
320	20.53	17.72	43.46	-25.75
330	20.55	17.80	44.10	-26.29
340	20.56	17.88	44.71	-26.83
350	20.57	17.96	45.31	-27.35
360	20.58	18.03	45.89	-27.85
370	20.59	18.10	46.45	-28.35

380	20.60	18.17	47.00	-28.88
390	20.60	18.23	47.53	-29.30
400	20.61	18.29	48.06	-29.77
410	20.62	18.35	48.56	-30.22
420	20.62	18.40	49.06	-30.66
430	20.63	18.45	49.55	-31.10
440	20.63	18.50	50.02	-31.52
450	20.64	18.55	50.48	-31.94
**$CH_3NH_3PbI_{3(s)}$**				
370	21.66	18.99	49.94	-30.95
380	21.66	19.06	50.52	-31.51
390	21.67	19.13	51.08	-31.95
400	21.68	19.19	51.63	-32.44
410	21.68	19.26	52.17	-32.91
420	21.69	19.31	52.69	-33.38
430	21.69	19.37	53.20	-33.83
440	21.70	19.42	53.70	-34.28
450	21.70	19.47	54.19	-34.72
